# “I miss my school!”: Examining primary and secondary school students’ social distancing and emotional experiences during the Covid-19 pandemic

**DOI:** 10.1007/s11125-022-09621-w

**Published:** 2022-10-12

**Authors:** Verena Letzel-Alt, Marcela Pozas, Christoph Schneider

**Affiliations:** 1 Section for Teacher Education and Research, University of Trier, Universitätsring 15, 54296 Trier, Germany; 2 Professional School of Education, Humboldt-Universität zu Berlin, Unter den Linden 6, 10099 Berlin, Germany; 3 School of Psychology, University of Monterrey, Monterrey, Mexico

**Keywords:** Social distancing, Covid-19, Students’ experiences, Homeschooling, Distance learning

## Abstract

With the rapid spread of Covid-19, countries around the world implemented strict protocols ordering schools to close. As a result, educational institutions were forced to establish a new form of schooling by implementing emergency remote education. Learning from home during the Covid-19 pandemic brought numerous changes, challenges, and stressors to students’ daily lives. In this context, major concerns have been raised based on the reports of students’ negative experiences resulting from social distancing and isolation. Given the impact of Covid-19 on many aspects of students’ lives, in particular their social and school experiences, research that provides insights into the consequences of this health crisis for students’ well-being has become important. This study aims to explore students’ experiences of social distancing and its relation to their negative emotional experiences during Germany’s first Covid-19–related school closure. Findings indicate that both primary and secondary students missed their friends and classmates and that primary school students perceived higher levels of social distancing. However, a linear regression analysis indicated that the older the students were, the more negatively affected they were by social distancing. The implications of the study’s results and further lines of research are discussed.

With the rapid spread of the Coronavirus (Covid-19), governments around the world imposed restrictions in order to reduce the risk of infection (UNESCO, 2020). Among these restrictions, social distancing became the most recommended measure, fundamentally affecting people’s lives (Greenhow & Chapman, 2020). Across media, social distancing was labeled as the new normal, while scientific literature defined it as the subjective perceived distance to a person or to members of a group (Beck, 2020). In the face of such a public health crisis, in which social distancing was compulsory, nearly all countries worldwide had to abruptly close their schools in March 2020. These immediate measures severely affected the education sector (Education International, 2020; Wyse et al., 2020), forcing educational institutions to establish a new form of schooling by implementing emergency remote education (ERE) (Bozkurt & Sharma, 2020). Students had to learn from home and utilize digital media to learn and study, while school administrators and teachers had to transition to an online environment within a very short amount of time (Wolff et al., 2020). Homeschooling, in the form of ERE during the Covid-19 pandemic can be defined as a form of distance learning organized by schools, supported by teachers, and accompanied by parents (Meyer, 2020). For a limited amount of time, an individual home-learning situation takes the place of learning in classrooms or learning groups. It is important to note that homeschooling as a response to the Covid-19 pandemic should not be confused with the concept of homeschooling widely known in the US, where parents voluntarily plan and organize their own children’s learning (Ladenthin, 2018).

Learning from home during the Covid-19 pandemic brought numerous changes, challenges, and stressors to students’ daily lives (Styck et al., 2021). The traditional school-based structure of learning, in which teachers are present to guide and support students, disappeared, and students’ daily routines were altered. Students were forced to organize their learning process themselves, which demanded self-regulated learning competences (Fischer et al., 2020). In addition, learning from home limited students’ interactions with their teachers, classmates, and friends to exchanges via online platforms (Wyse et al., 2020).

The term *social distancing* has been used frequently in the context of the Covid-19 pandemic. However, its theoretical construct and operationalization are still being discussed and developed. Conventionally, social distancing can be understood as maintaining physical distance from other people (Abel & McQueen, 2020; Aminnejad & Alikhani, 2020). For the purpose of the present study, however, social distancing is defined as the subjective, perceived distance to another person or to members of a group (Beck, 2020). Current research conducted during the Covid-19 pandemic lockdowns reports that social distancing can have a negative impact on mental health in the form of conditions such as depression, anxiety, and loneliness (Abel & McQueen, 2020; Armbruster & Klotzbücher, 2020; Xie et al., 2020). Despite social distancing, however, people can remain in contact using digital devices for chatting, telephoning, and video calling. Moreover, a stronger contact with one’s own family may counterbalance the missing physical closeness to others (Pozas et al., 2021; Adami et al., 2020; Saad & Gupta, 2020). Flack et al. (2020) highlighted that teachers consider their students to be socially isolated as a result of ERE during the pandemic. Additionally, the authors reported that perceptions among primary school teachers in particular are that the emotional experience of social distancing affects students' academic performance. (López et al., 2017). According to Schreiber and Jenny (2020), emotional experiences can be defined as positive and negative affective activations. A high level of activation is crucial for an affective activation to be considered either positive or negative. Being highly motivated, for example, can be considered a high activation state with a positive affect, whereas being nervous or worried are examples of a high activation state with a negative affect (Schreiber & Jenny, 2020). According to Huber and Helm (2020b), a majority of students indicated that they experienced stress due to distance learning; however, most students did not name specific challenges. It is important to mention that Huber and Helm (2020b) took into account only learning challenges and not those with regard to social distancing.

Recent research on both parents’ and students’ experiences during the Covid-19 crisis has shown that social distancing has provided families with both positive and negative experiences. On the one hand, because parents had to work from home, parents and children felt that time spent together as a family was a positive aspect of the shutdown (Pozas et al., 2021). Furthermore, studies focusing solely on students’ experiences during the Covid-19 school closures have shown that students found that working on their own and planning their learning according to their own educational needs was beneficial (Holtgrewe et al., 2020). On the other hand, major concerns have been raised based on reports of students’ negative experiences (Flack et al., 2020). A recent study by Styck et al. (2021) indicated that not meeting friends in person was the greatest stressor among elementary, middle, and high school students in the United States. This result is in accord with scientific literature pointing out that remote education may evoke feelings of disconnection (Smith & Taveras, 2005). Moreover, studies by Xie et al. (2020) and Zhou et al. (2020) suggest that students are experiencing significant levels of anxiety and depressive symptoms due to social isolation during the lockdown. A detailed study by Demaray et al. (2021) indicated that there were gender differences regarding anxiety and depression symptoms associated with stressors due to Covid-19, with an alarmingly high level of depressive symptoms for high school female students. Similarly, Karasmanaki and Tsantopoulos (2021) and Styck et al. (2021) reported that female participants experienced more stress in comparison to their male counterparts, especially when it came to fears of social distancing (Fedorenko et al., 2020). In sum, it can be assumed that student experiences during social distancing (or isolation) from school are particularly relevant in the current homeschooling context (Brooks et al., 2020; Demaray et al., 2021; Flack et al., 2020; Kira et al., 2020; Styck et al., 2021; Taylor et al., 2020).

Given the far-reaching impact of Covid-19 on many aspects of students’ lives, in particular their social and school experiences, research is of the utmost importance for providing insights into the impact of this health crisis on students’ emotional well-being (Huber & Helm, 2020a) and for supporting the development of intervention measures (Styck et al., 2021). More research should provide a better understanding of the specific characteristics of individuals and contexts that can have potentially negative outcomes on students (Demaray et al., 2021). A fair amount of recent research has reported on challenges and stressors, such as lack of resources, family conflict, social distancing, and fear of illness. However, given the importance of a child’s microsystem, such as the school context (Demaray et al., 2021), the concrete experiences of children while physically distanced from their school should also be the subject of research. Moreover, research specifically focused on disasters and health crises has shown that it is important to consider gender and age (and educational stage) differences with regard to impacts (Brock et al., 2016; Spagnolo et al., 2020). Hence, the present study aims to determine if there were significant differences in students’ experiences of social distancing according to gender and educational stage (primary and secondary school) as well as variations in the impact of social distancing on students’ emotional experiences. The following research questions led the data analyses:

1. How do students experience social distancing with regard to their school life?
2. Are there differences across gender and educational stage with regard to the experiences of social distancing?
3. How are students’ experiences of social distancing related to their emotional experiences?

## Methods

### Participants

The data for this study were collected in Germany in a project known as Student-Parents-Teachers in Homeschooling (SCHELLE, following the German term Schüler/-innen-Eltern-Lehrkräfte) (see Letzel et al., 2020). The data were collected from an online survey taken from April to June 2020, coinciding with the first lockdown of the Covid 19 pandemic. Students had to provide their parents’ or tutors’ consent before accessing the online survey. The sample consisted of 150 students (62% female) with a mean age of 15.27 years. Grouped according to educational stage, 6.7% of the students were from primary school (grades one to four) and 93.3% from secondary school (grades five to thirteen). To the best of our knowledge, there was currently no existing, evaluated, and validated scale that fit the research questions in this study. Therefore, the authors decided to develop a scale based on previous literature. The student experiences of social distancing scale see (see Table 1 for the German -language version and English translation) was developed based on the work of Beck (2020) and Bronfenbrenner’s Process-Person-Context-Time model (Tudge et al., 2009), from which important components were identified. These include:

- Proximal processes (reciprocal interactions between a developing human being and one or more of the persons, objects, and elements in his or her immediate environment)
- The microsystem (the immediate setting in which the developing human can engage in proximal processes, in this case, the school)
- The macrotime (events in the larger society within and across generations that affect or are affected by processes and outcomes of human development over the life course, in this case the Covid-19 pandemic crisis).

**Table 1:** Means, standard deviations, t-statistics, and effect size

Item	*M*	*SD*	*t*(147)	*p*	Cohen’s *d*
1. I miss my friends.	3.65	.74	18.82	< .001	.73
2. I miss my classmates.	3.31	.90	10.91	< .001	.90
3. I miss the school itself.	2.67	1.11	1.90	n.s.	–
4. I miss my teachers.	2.46	1.01	− .53	n.s.	–

After intensive literature study and in cooperation with representatives from science, schools, and educational planning, four items were constructed. It was decided to use a 4-point Likert scale (1= I do not agree at all to 4 = I fully agree) to avoid the potential of a tendency to the middle (Jonkisz et al., 2012). The scale was introduced with a short instruction asking students to indicate how they evaluate and perceive their current situation at school given the Covid-19 school closure (“Think about your experiences during the Covid-19 homeschooling situation. Please check the response that most represents your experience”.). The items were developed in German and translated into English for this publication.

Exploratory Factor Analysis (EFA) was carried out for the four items using principal components analyses with varimax rotation. The Kaiser-Guttman criterion (retain all factors with eigenvalues greater than 1.0) was used to identify the number of factors (Osborne & Costello, 2009). The Kaiser-Meyer-Olkin value was .65, exceeding the recommended value of .06 (Tabachnick & Fidell, 2007), and Bartlett’s test of sphericity reached statistical significance (χ^2^ (6) = 180.15, *p* < .001), indicating that there was sufficient communality in the manifest variables and that the data are suitable for the factor analysis (Field, 2013; Pallant, 2010). The EFA with the 4 items yielded one single factor accounting for 58.04% of the variance. Table 1 shows the factor loading of the four items on the single factor named as ‘Student Experiences of Social Distancing’. The reliability of the four-item scale was α = .75. The inter-item correlation ranged from .47 to .64. Although the inter-item correlations for two items are relatively moderate, the reliability of the scale did not improve by removing them. Hence, all four items were kept in the scale.

To assess students’ positive and negative activation, the short-form scale for positive activation, negative activation, and valence (PANAVA) scale was used (Schallberger, 2005). The PANAVA scale measures students’ positive and negative activation by asking the participants to indicate their emotional state on a 7-point scale consisting of eight items of opposite adjective pairs, with four items representing the positive activations and four the negative activations (Schreiber & Jenny, 2020). Positive activation is represented by the adjective pairs “no energy–full of energy”, “tired–wide awake”, “listless–highly motivated”, and “bored–enthusiastic”, with the first adjective expressing a state of low positive activation and the second a state of high positive activation. The items illustrating negative activation are: “relaxed–stressed”, “peaceful–angry”, “calm–nervous”, and “free of worry–worried”. In each case, the first adjective indicates an emotional state of low negative activation and the second an emotional state of high negative activation (Schallberger, 2005). Previous research gives evidence that the PANAVA scales possess acceptable internal consistencies. (For the positive activation scale, the value of Cronbach’s alpha is α = .86, and for the negative activation scale, α = .86). For the sample of this study, Cronbach’s alpha for the positive activation was α = .81, and for the negative activation scale α = .78. The students’ reports on their positive and negative activation before distance learning occurred in retrospectively, since the data collection took place during distance learning.

### Data analysis

The analyses within this study were conducted using SPSS 27. In order to get detailed insights into research question 1 (the students’ experiences of social distancing) one-sample t-tests were calculated separately for the social distancing scale’s four items. Concerning research question 2, gender differences were analyzed using an independent sample t-test in which, given the imbalance in sample size between primary and secondary students, education -stage differences were explored through a Mann-Whitney nonparametric test (Field, 2013). Finally, in order to examine research question 3, two multiple linear regression analyses were performed and students’ emotional experiences of positive and negative activation were calculated.

## Results

One sample *t* test on each item (based on a scale of 1 to 4 where the theoretical mean is 2.5) was calculated. From Table 1 it can be seen that items 1 and 2 were rated higher by the sample, whereas items 3 and 4 were not statistically significant. The effect size of item 1 is large, while that of item 2 is medium, according to Cohen’s d results, where an effect size of 0.20 is small, 0.50 is medium, and 0.8 or above is large (Cohen, 1988). Item 2, “I miss my classmates”, had the highest effect size (d = .90), followed by item 1, “I miss my friends” (d = .73).

Means, standard deviations, and correlations of all variables under study can be found in Table 2. A one -sample *t *test was conducted to explore at a scale-level students’ general social distancing experience. Given that the empirical answer score is above the theoretical mean value of the scale (M = 2.5) (t(146) = 8.82, *p* < .001), the findings indicate that students perceive a high level of distance and separation from their peers, teachers, school, and friends. The results of an independent t-test show that female and male students have a similar perception of social distance (t(144) = − .38, n.s.). Given that more than 90% of the respondents were in secondary school, it was decided to explore potential differences between primary and secondary school students using the Mann-Whitney nonparametric test (Field, 2013). The results show that primary school students perceive a higher degree of social distance (Mdn = 3.00) than secondary school students (Mdn = 2.00), U = 231, z = − 2.95, r = − .25.

**Table 2:** Factor loadings and inter-item correlation of the items on the students’ experiences of social distancing scale (N = 150***)***

Item		Factor loadings	Inter-item correlation
English version	German version		
1. I miss my classmates.	Ich vermisse meine Klassenkameraden/-innen	.85	.64
2. I miss the school itself.	Ich vermisse die Schule an sich.	.79	.49
3. I miss my friends.	Ich vermisse meine Freunde.	.72	.61
4. I miss my teachers.	Ich vermisse meine Lehrkräfte.	.68	.47

The first multiple regression model was conducted to see if age, gender, educational stage, and social distancing level predicted students’ positive activation during the Covid-19 related homeschooling. As seen from Table 3, the analyses revealed that both students’ age and social distancing negatively predict their positive activation. It appears that the younger the students are and the less social distancing they experience, the higher their positive activation.

**Table 3:** Means, standard deviations, and correlations of all variables

	M	SD	1	2	3	4	5	6
1. Age	15,27	3.37	–					
2. Gender	–	–	.25**	–				
3. Educational stage	–	–	.59**	.22*	–			
4. Social distancing	3.02	.71	− .36**	.03	− .23**	–		
5. Positive activation during homeschooling	3.59	1.43	− .02	− .04	.01	− .39 **	–	
6. Negative activation during homeschooling	3.94	1.53	.16	− .17*	− .04	.30**	− .66**	–

*p < .05; **p < .01

The second multiple regression model was calculated to explore whether age, gender, educational stage, and social distancing level predicted students’ negative activation during the Covid-19 related homeschooling. The results show again that students’ age and the experienced social distancing predicted their negative activation. However, as seen from Table 3, this is opposite to the previous model, meaning that the older the students are and the higher the levels of social distancing they experience, the more negative activation occurred.

## Discussion

The results of this study give a first impression of how students experienced social distancing from their school, teachers, and classmates as well as the impact it had on their emotional experiences. With regard to the item-level analyses, students rated significantly higher those items related to missing their friends and classmates. This result goes in line with findings from Styck et al. (2021) in the United States and Heidrich et al. (2022) in Austria, where students also reported not seeing their friends and classmates as the highest stressor during the Covid-19 school shutdown. However, it is important to highlight that in the present study the item ‘I miss my classmates’ had the largest effect size. Thus, these results strengthen the argument that the school context is a crucial microsystem for children and youth, particularly during the current Covid-19 crisis (Demaray et al., 2021).

Furthermore, the findings from this study reported no significant differences across students’ gender. Hence, both female and male participants missed school and their peers in a similar way. This result is similar to the findings of Heidrich et al. (2022) with an Austrian student sample but contradicts findings from studies such as the one from Fedorenko et al. (2020) that clearly indicated gender differences. For instance, Fedorenko et al. (2020) found that female participants reported higher levels of fear of social distancing. However, the discrepancy between the studies may stem from the country and surveyed sample context (United States vs. Germany) and the different research focus. Further research is needed to explore in detail these contrasting results in order to determine whether country -specific or other variables come into play (Table 4).

**Table 4:** Multiple regression models

Variable	Positive activation	Negative activation
	β	β
Age	− .21^+^	.33**
Gender	.15	− .01
Educational stage	− .02	− .12
Social distancing	− .45**	.40**
R2	.20**	.19**

^+^*p* <.10; *p < .05; **p < .01

Consistent with the findings of Heidrich et al. (2022), the data analyses indicated that younger students (those in primary school) experienced high levels of social distancing. Previous research has also raised concerns about the potential negative impact of social distancing on the well-being of primary school students (Flack et al., 2020). The present study seems consistent with such conclusions, since primary school students clearly rated a higher level of distance, loneliness, and disconnection from their peers, teachers, and school context.

Finally, with regard to the results obtained from the multiple linear regression, it can be concluded that social distancing has a critical impact on students’ emotional experiences. A manifold of research has widely discussed the negative impact of social distancing on children and adolescents. For instance, Ellis et al. (2020) found that students’ stress over social distancing during the Covid-19–related closures was strongly associated with increased feelings of loneliness and depression. However, as seen from the correlation analyses and both multiple linear regression models, older students appear to be more negatively affected by social distancing. That is, the older the students are, the lower their positive activation and the higher their negative activation as a result of social distancing. Similar findings reported by Wang et al. (2021) have revealed that social distancing predicted an increase in adolescents’ stress and decreases in their positive activation.

## Limitations and considerations for further research

Underlying this study are several limitations that must be considered. First, given the sample size (N=150), it was not possible to establish two samples in order to carry out confirmatory factor analyses as well as validation procedures (i.e., criterion, construct, and content validity) (Svensson, 2011). However, because of the unprecedented situation presented by the pandemic, there were no available, well-established instruments that could be implemented. Second, the number of primary school students is relatively low compared to the number of secondary students. Therefore, the results of this study must be considered with caution. Nonetheless, the decision to compare educational stages was derived from available international research that has pointed out differences between primary and secondary school with regard to the effects of social isolation (Demaray et al., 2021; Flack et al., 2020; Styk et al., 2020). Third, the study used data collected in Germany; thus, generalizing the results to other countries is problematic as differences across countries can be identified only when research follows a cross-country approach and uses the same instruments. Given the features of the scale we used (it is short, reliable, and easy-to-administer), it has the potential to be used internationally, as well, following other countries’ respective back translations and validation studies. This would provide insights that help us better understand potential contextual differences, as in Fedorenko et al. (2020). Finally, the data stem from a cross-sectional study; it would be desirable to use the scale in a longitudinal study that would also provide insights into the development of students’ social distancing experiences, as the pandemic is a phenomenon that students around the world may encounter in the future.

## Conclusions

Despite its limitations, the present study highlights the severe impact of social distancing on students’ emotional experiences and well-being. Students’ experience a drastic reduction in their social interactions during Covid-19 school-related closures. This reduction appears to challenge their need for relatedness in ways that have contributed to higher levels of negative affect and reduced positive activation. Given that the Covid-19 pandemic is still and will most likely continue to be quite an unpredictable situation, it is of upmost importance for schools and teachers to support students’ social interaction. Lastly, the study’s results only strengthen what Bozkurt and Sharma (2020, p. 106) conclude: “Perhaps, one of the lessons we must draw from this experience is that no amount of technology is able to replace face-to-face interaction or the care and emotion present in a classroom”.
